# Zoom fatigue related to online learning among medical students in Thailand:  Prevalence, predictors, and association with depression

**DOI:** 10.12688/f1000research.146084.2

**Published:** 2024-08-27

**Authors:** Veevarin Charoenporn, Sirashat Hanvivattanakul, Kanathip Jongmekwamsuk, Rinradee Lenavat, Korravit Hanvivattanakul, Thammanard Charernboon

**Affiliations:** 1 Department of Psychiatry, Faculty of Medicine, Thammasat University, Pathumthani, Thailand; 2 Department of Psychiatry, Thammasat University Hospital, Pathumthani, Thailand; 3 Faculty of Medicine, Thammasat University, Pathumthani, Thailand; 4 Faculty of Medicine, Chulalongkorn University, Bangkok, Thailand; 5 College of Dental Medicine, Rangsit University, Pathumthani, Thailand; 6 Department of Clinical Epidemiology, Faculty of Medicine, Thammasat University, Pathumthani, Thailand

**Keywords:** depression, medical students, online learning, videoconferencing fatigue, Zoom fatigue

## Abstract

**Background:**

Amidst the COVID-19 pandemic, the learning pattern of medical students shifted from onsite to online. This transition may contribute to what has been called “Zoom fatigue.” This study aimed to evaluate the prevalence of Zoom fatigue related to online learning, identify associated factors of Zoom fatigue, and explore its correlation with depression among medical students during the COVID-19 pandemic.

**Methods:**

This cross-sectional study was conducted among 1st to 6th-year Thai medical students. The online survey was administered using a demographic and health behavior questionnaire, the Patient Health Questionnaire-9 (PHQ-9), and the Thai version of the Zoom Exhaustion & Fatigue Scale (ZEF-T).

**Results:**

Among the 386 participating students, 221 (57%) were female, with a mean age of 20.6 years. The prevalence of high Zoom fatigue was 9.6%. In the multivariable regression analysis, a lower academic year and a higher number of online learning sessions were significant predictors of Zoom fatigue (p < 0.001), while regular exercise emerged as a protective factor (p = 0.009). The prevalence of depressive disorder was 61.9%, and a significant correlation was found between having a depressive disorder and experiencing Zoom fatigue (p = 0.004).

**Conclusion:**

Zoom fatigue among medical students was correlated with depression. Consequently, medical students experiencing Zoom fatigue should undergo further assessment for depression. It is crucial to closely monitor medical students in lower academic years with a high number of online sessions for signs of Zoom fatigue. Additionally, implementing strategies, such as reducing the frequency of online sessions and promoting regular exercise, may help alleviate the symptoms.

## Introduction

Since the start of 2020, COVID-19, caused by the SARS-CoV-2 virus, has quickly spread worldwide, becoming a major global health crisis. This pandemic has led to unpredictable and mandatory transformations on a global scale, profoundly impacting the lives of individuals worldwide. ^1^ The WHO has recommended implementing several preventive strategies, such as social distancing measures, restrictive regulations, self-quarantine, and transitioning to remote work, to reduce the spread of the COVID-19 pandemic and minimize mortality rates. ^2^ In the realm of education, these changes have contributed to the new normal lifestyle where face-to-face and onsite learning have become impractical. Online lectures have become the standard, common, and fostering the widespread use of online meeting applications like Google Classroom, Google Meet, Zoom, Webex, and Microsoft Teams as major platforms for online learning. ^3^

Zoom emerged as one of the most popular videoconferencing platform for online learning during the COVID-19 pandemic. It is used for both personal and business purposes. The number of daily users skyrocketed from 10 million in December 2019 to 300 million in April 2020, making it the fastest-growing application in 2020. ^4^ However, excessive use of videoconferencing can lead to the novel phenomenon called “Zoom fatigue” or “Zoom exhaustion,” defined as physical and mental exhaustion from participating in virtual meetings through any online meeting application. ^5^ This condition may manifest in several domains, including general, emotional, visual, motivational, and social fatigue. ^6^

The study postulated the phenomenon of Zoom fatigue, linked to the excessive use of nonverbal communication in video conferencing. In comparison to regular meetings, video conferencing requires heightened concentration and attention. Various factors contribute to Zoom fatigue, including eye strain from prolonged close-range staring, increased cognitive load processing information in video calls, self-consciousness from constant self-reflection during calls, and restricted body movements in online sessions. Additionally, external factors beyond one’s control, such as internet disruptions, the pressure to respond to speaker questions, and environmental conditions, can exacerbate Zoom fatigue. Therefore, the condition of Zoom fatigue may impact individuals’ overall well-being. ^7^

A specific assessment tool to measure video-related fatigue, called the Zoom Exhaustion and Fatigue (ZEF) scale, was developed at Stanford University in 2021. ^6^ It comprises 15 items and assesses five domains: general fatigue, visual fatigue, social fatigue, motivation fatigue, and emotional fatigue. The scale has been translated into several languages. ^8^^,^^9^

Medical students have faced profound challenges due to the COVID-19 pandemic and the transition to online learning. In a cross-sectional study conducted at a Brazilian medical school using the ZEF scale, findings revealed that up to 56% of participants experienced high levels of Zoom fatigue. ^10^ Beyond Zoom fatigue, studies also indicate a substantial increase in depression, anxiety, and mental problems, especially among university students, during the COVID-19 pandemic. ^11^^–^^14^ These findings suggest that university students, particularly medical students, are among the populations most affected by the mental health consequences of the pandemic, facing a heightened risk of developing depression and psychological problems. ^14^

In addition, several studies have demonstrated the relationship between Zoom fatigue and mental health conditions. A national survey conducted in the United States during the pandemic found a link between Zoom fatigue and depressive symptoms. ^15^ Another study also showed a positive correlation between Zoom fatigue and depression, anxiety, and stress, while showing a negative correlation between Zoom fatigue and life satisfaction and academic well-being. ^16^

Due to its relatively recent emergence, Zoom fatigue has been the subject of limited research. studies specifically focusing on medical students are even scarcer. Medical students differ significantly from other student populations, undergoing a six-year curriculum that includes clinical practice and often demanding on-call shifts. Understanding the risk factors for Zoom fatigue in this population could inform tailored prevention strategies and potentially mitigate negative impacts on academic performance and clinical decision-making.

During the COVID-19 pandemic, all lectures at our university were delivered online, exposing the entire medical student body to this learning modality. To our knowledge, there are still only a few studies examining the prevalence of Zoom fatigue, its risk factors, and its potential association with depression, with no such study conducted among medical students in Thailand. To fill this knowledge gap, our study aims to assess the prevalence of Zoom fatigue in medical students during the COVID-19 pandemic, identify associated potential risk factors, and investigate its correlation with depression.

## Methods

A cross-sectional online survey was conducted among medical students at Thammasat University, Thailand, from January to July 2022. This study received approval from the Human Research Ethics Committee of the Faculty of Medicine, Thammasat University (number 028/2565; date of approval: January 26, 2022), in accordance with the Declaration of Helsinki. Signed informed consent was waived by the Ethics Committee due to the online nature of the survey; however, participants were provided with study information on the first page.

### Participants

The recruitment process involved 386 medical students from Thammasat University. To meet the inclusion criteria, participants had to be Thammasat medical students aged at least 18 and proficient in Thai. They were requested to complete an anonymous online survey questionnaire using Google Forms, which was distributed through various online platforms such as LINE, university forums, and an exclusive Facebook group for Thammasat medical students. The survey tool automatically verified that all questions were completed before submission; therefore, our study had no missing data. No compensation or incentives were offered to participants. Confidentiality and privacy of data were ensured throughout the study process.

### Measures

The questionnaire comprised demographic data, the Thai version of the Patient Health Questionnaire (PHQ)-9, and the Thai version of the Zoom Exhaustion and Fatigue Scale (ZEF-T).

*Demographic Data*

The basic general information includes gender, age, academic year, underlying disease, the number of online sessions per day, duration of each online session, time taken for each break during online sessions, exercise frequency, sleep problems, and experience of failing an examination.

*The Zoom Exhaustion & Fatigue Scale (ZEF)*

The ZEF is a questionnaire comprising 15 items that assess the symptoms experienced by participants during video conferences. The questions are categorized into five domains, including general fatigue, visual fatigue, social fatigue, motivation fatigue, and emotional fatigue. Each question on the questionnaire is scored from 1 to 5 (1 = not at all, 2 = slightly, 3 = moderately, 4 = very, 5 = extremely), resulting in a total score ranging from 15 to 75 points. A higher score indicates a higher level of Zoom fatigue. ^6^^,^^8^ The average total ZEF score is calculated by dividing the total ZEF score by 15. We established a cutoff score for Zoom fatigue as an average total ZEF score of ≥4 to identify individuals experiencing Zoom fatigue. The ZEF was granted permission to be used and translated by Fauville G. The Thai version of the ZEF was developed using forward and backward translation. The details and other psychometric properties are presented in Charoenporn and Charernboon (2023). ^8^

*The Patient Health Questionnaire (PHQ-9)*

The Patient Health Questionnaire (PHQ-9) is a self-assessment tool comprising nine questions designed to evaluate the frequency of symptoms associated with depression over a two-week period. The questionnaire employs a rating scale ranging from 0 to 3 points, with categories including none (0 points), some days but not often (1 point), quite often (2 points), and almost every day (3 points). The total score obtained ranges from 0 to 27 points, with a higher score indicating more severe depressive symptoms. ^17^ In this study, we applied a cutoff score of ≥9 points, based on the PHQ-9 Thai version, to identify individuals experiencing depression. ^18^ PHQ-9 is publicly available, and no permission is required to use, reproduce, or distribute the tools. https://www.apa.org/depression-guideline/patient-health-questionnaire.pdf.

### Statistical analyses

The sample size was determined using the infinite population proportion formula, with proportion = 0.48, ^10^ error (d) = 0.05, alpha (α) = 0.05, and Z = 1.96. The calculated sample size was determined to be at least 384 students. Participant characteristics were presented using descriptive statistics. Multivariable linear regression analysis was employed to examine the factors associated with Zoom fatigue scores. Exact tests were utilized to analyze the association between depression and Zoom fatigue. All statistical calculations were performed using STATA Version 14.0 (StataCorp LLC, College Station, TX, USA), with statistical significance set at a p-value less than 0.05.

## Results

### Demographic data and possible associated risk factors

Most participants were female (57.3%), with a mean age of 20.6 years. The majority were third-year medical students (29.3%), followed by first-year (26.4%). About 18.4% of participants reported having an underlying disease. The average number of Zoom sessions per day was 1.9, with 83.7% of participants having sessions lasting one hour or more. Regarding sleep, 56.0% of participants reported not getting enough sleep, and 62.2% reported exercising sometimes. ( Table 1).

**Table 1.** Demographic data.

Variables (n = 386)	N (%)
Gender: female	221 (57.3%)
Age: mean (SD)	20.6 (2.0)
Education year	
1	102 (26.4%)
2	54 (14.0%)
3	113 (29.3%)
4	64 (16.5%)
5	33 (8.6%)
6	20 (5.2%)
Underlying disease	71 (18.4%)
Number of zoom session per day: mean (SD)	1.9 (1.0)
Duration of zoom session per session	
- <1 hour/session	63 (16.3%)
- ≥1 hour/session	323 (83.7%)
Break time during each session	
- 0-30 minutes	158 (40.9%)
- >30 minutes	228 (59.1%)
Sleep	
- enough	170 (44.0%)
- not enough	216 (56.0%)
Exercise	
- no	88 (22.8%)
- sometimes	240 (62.2%)
- regular	58 (15.0%)
Had failed an exam	
- no	194 (50.3%)
- yes	192 (49.7%)

### Zoom Exhaustion and Fatigue (ZEF) score

Table 2 shows the mean total ZEF score and its subscales, including general fatigue, visual fatigue, social fatigue, motivation fatigue, and emotional fatigue. The average total mean score for the ZEF was 2.8 indicating a slight level of Zoom fatigue. The highest subscores were observed in the general fatigue and social fatigue domains at 9.2 points, while the emotional fatigue had the lowest score at 7.3.

**Table 2.** Zoom Exhaustion and Fatigue (ZEF) total score and subscale scores.

	Mean (SD)
General fatigue	9.2 (3.0)
Visual fatigue	7.9 (3.2)
Social fatigue	9.2 (3.1)
Motivational fatigue	8.9 (2.9)
Emotional fatigue	7.3 (2.9)
Total ZEF score	42.5 (12.5)
Average total ZEF score	2.8 (0.83)

### Prevalence of Zoom fatigue

Table 3 presents the levels of Zoom fatigue. The majority of participants (54.1%) reported experiencing no or only a slight level of Zoom fatigue. The overall prevalence of Zoom fatigue among participants was found to be 9.6%, categorized as a very/extremely high level of Zoom fatigue, with an additional 36.3% experiencing a moderate level of Zoom fatigue.

**Table 3.** Prevalence of zoom fatigue.

Average total Zoom Exhaustion and Fatigue score	N (%)
1-1.99 (not at all)	63 (16.3%)
2-2.99 (slightly)	146 (37.8%)
3-3.99 (moderately)	140 (36.3%)
≥4-4.99 (very/extremely)	37 (9.6%)

### The relationship between Zoom fatigue and depression

The prevalence of depression among the participants was 61.9% (n = 239). The results reveal a statistically significant relationship between having depression and Zoom fatigue (p = 0.004). Participants experiencing Zoom fatigue had a higher prevalence of depression compared to those without Zoom fatigue (83.8% vs 59.6%) ( Table 4).

**Table 4.** Association between zoom fatigue and depression.

	Depression		Total
Zoom fatigue	Yes	No	
**Yes**	31 (83.8%)	6 (16.2%)	37 (9.6%)
**No**	208 (59.6%)	141 (40.4%)	349 (90.4%)
**Total**	239 (61.9%)	147 (38.1%)	386 (100%)

Exact test p = 0.004.

### Factors associated with Zoom fatigue

Table 5 presents a multivariable linear regression analysis demonstrating factors associated with the ZEF total scores. The analysis found that education year, regular exercise, and the number of Zoom sessions per day were significantly associated with total ZEF scores. Specifically, a lower education year was associated with higher ZEF scores (coefficient = -2.44, p < 0.001), while regular exercise was associated with lower ZEF scores (coefficient = -5.27, p = 0.009). Conversely, a higher number of Zoom sessions per day was associated with higher ZEF scores (coefficient = 2.72, p < 0.001). Gender, age, underlying disease, not getting enough sleep, duration of breaks between sessions, and having failed an exam were not found to be statistically significant factors associated with ZEF scores.

**Table 5.** Factors associated with Zoom Exhaustion and Fatigue (ZEF) total scores using multivariable linear regression analysis.

Variable	Coefficient	p-value
Gender: female	2.27	0.061
Age (year)	0.46	0.231
Education year	-2.44	<0.001
Underlying disease	2.94	0.057
Exercise		
- no	Ref	Ref
- sometimes	-1.36	0.353
- regular	-5.27	0.009
Sleep: not enough	2.39	0.053
Had failed an exam	-0.51	0.674
Number of zoom session/day	2.72	<0.001
Break time during each session		
- >30 minutes	Ref	Ref
- 0-30 minutes	-0.54	0.655

## Discussion

To the best of our knowledge, this study represents the first investigation in Thailand into the prevalence of Zoom fatigue, associated factors, and its correlation with depression among Thai medical students during the COVID-19 pandemic. The results of the study reveal a 9.6% prevalence of Zoom fatigue among Thai medical students as a consequence of online learning during the pandemic. Furthermore, the study uncovers a significant relationship between Zoom fatigue and depression, emphasizing the importance of addressing both conditions, as they may impact students’ well-being and overall quality of life. ^15^

Our study revealed that the occurrence of Zoom fatigue among medical students was significant and supports the idea that it is a common phenomenon among individuals using online platforms for learning. The high prevalence may be explained by the implementation of social distancing measures throughout the country, with the university mandating the use of online learning for all lectures and restricting onsite sessions. This led to the necessity of attending at least two online sessions per day, each averaging over an hour per session. These online sessions demanded prolonged concentration, extended close-range staring, and a high cognitive load in learning and using videoconference programs. ^5^^,^^6^

The prevalence in our study aligns with previous research conducted during the COVID-19 pandemic, which demonstrated moderately to high Zoom fatigue ranging from 48% to 68.6%. ^10^^,^^19^ Slight variations may be attributed to various factors such as videoconferencing time, study faculty, study type (hybrid or online only), and the type of online learning (lecture or more interactive meeting). Another crucial factor is that Zoom fatigue is a relatively new concept, lacking a worldwide standard criteria like major depressive disorder in DSM-5 or ICD-10. Therefore, the definition may vary between studies and depend on the questionnaires used. Consequently, it might be challenging at this time to directly compare the prevalence of Zoom fatigue between studies.

The study identified a significant correlation between Zoom fatigue and depression, with a substantial number of participants experiencing both Zoom fatigue and depression. Consistent with our findings, other studies, such as those by Elbogen et al. and Montag et al., also reported similar findings, indicating that depressive symptoms exhibited a significant association with Zoom fatigue, even when adjusting for demographic, psychosocial, and clinical covariates. ^15^^,^^20^ The connection between these two conditions might be explained by the overlapping symptoms observed in both Zoom fatigue and depression. For instance, social fatigue translates into an unwillingness to engage with others after videoconferences, while motivation fatigue reflects a diminished drive following such sessions. These symptoms bear a striking resemblance to the loss of interest commonly seen in major depressive disorders. Moreover, irritability and moodiness can also be found in depressive disorders. The connection between Zoom fatigue and depression may also be elucidated by the social isolation and loneliness associated with both conditions. Higher videoconference use might indicate increased social isolation and loneliness, contributing to the development or exacerbation of depression. Additionally, several studies have indicated that Zoom fatigue is linked not only to depression but also to psychological distress and lower life satisfaction. ^16^

This finding is unsurprising and aligns with the majority of previous studies that have demonstrated a correlation between an increased number of video conferencing sessions and higher fatigue. ^6^^,^^19^^,^^21^ Our study also identified lower academic years as another notable risk factor. This observation may be attributed to the relatively greater number of lecture hours in the preclinical years (1st – 3rd year) compared to the clinical years (4th – 6th year). Clinical medical students dedicate a substantial amount of time to engaging with patients in hospitals and participating in hands-on learning activities, including basic skill workshops and bedside teaching. Their education primarily focuses on real patient cases encountered in a hospital setting, rather than relying on theory-based lectures. Even during the pandemic, clinical students continue to work in hospitals and participate in bedside teaching, resulting in lower video conferencing usage. On the other hand, preclinical students mainly learn through lectures. Therefore, the challenges brought about by the transition to online learning during the pandemic may have affected preclinical students more than clinical students. This is consistent with previous studies conducted on both medical and non-medical students, which revealed that students in the lower academic years were more susceptible to experiencing mental distress related to their studies. ^22^

Regular exercise has been identified as a protective factor against Zoom fatigue, suggesting that students consistently engaging in physical activity may exhibit better resilience to online learning challenges, reducing the likelihood of experiencing Zoom fatigue. This association can be explained by considering one of the underlying causes of Zoom fatigue—limited mobility or constrained body movements. Regular exercise promotes increased body movement, potentially mitigating the onset of Zoom fatigue. ^7^ Furthermore, compelling evidence indicates that exercise prevents various mental disorders, such as depression and anxiety disorders, offering multiple beneficial effects on both physical and mental health. Therefore, exercise may also play a role in preventing Zoom fatigue. ^23^

### Strength and limitations

This study represents the initial exploration of the adverse effects of online learning on medical students in Thailand, revealing a potential connection between Zoom fatigue and depression. However, certain limitations must be acknowledged. Participants were recruited from a single site, which may limit the generalizability of the findings, and the results might not be applicable to other faculties. The cross-sectional nature of the study prevents the establishment of a causal relationship. Additionally, the reported prevalence might be underestimated due to voluntary sample collection, potentially excluding highly fatigued students from survey participation. The novelty of the concept of Zoom fatigue has resulted in relatively limited studies on this topic, leading to a lack of standardized criteria in various studies, which hampers direct prevalence comparisons. Lastly, the study did not clearly differentiate between the use of online meeting programs for lectures or interactive meetings. Further investigations are recommended to distinguish Zoom fatigue arising from online lectures and meetings distinctly.

## Conclusion

To date, the use of video conferencing in education persists beyond the pandemic, becoming a part of our new normal lifestyle. It is imperative for instructors and users to recognize Zoom fatigue as a potential negative consequence. The significant prevalence of Zoom fatigue among medical students, along with its correlation with depression, emphasizes the importance of screening for both conditions, especially in lower academic years. Implementing proactive strategies, such as reducing session lengths and frequency, and promoting regular exercise, may contribute to symptom prevention.

### Ethics and consent

This study received approval from the Human Research Ethics Committee of the Faculty of Medicine, Thammasat University (number 028/2565; date of approval: January 26, 2022), in accordance with the Declaration of Helsinki. Signed informed consent was waived by the Ethics Committee due to the online nature of the survey; however, participants were provided with study information on the first page.

## Data Availability

The data supporting the findings of this study are available on Figshare: “Zoom fatigue among medical students” at https://doi.org/10.6084/m9.figshare.25407883. This project contains the raw data of the study. Data are available under the terms of the Creative Commons Attribution 4.0 International license (CC-BY 4.0). A copy of the Thai version of the ZEF questionnaire used in the study is available on Figshare: “Zoom Exhaustion & Fatigue Scale - Thai Version (ZEF-T)” at https://doi.org/10.6084/m9.figshare.25407931. The original English version of the ZEF can be accessed from Fauville G, Luo M, Queiroz ACM, Bailenson JN, Hancock J. Zoom Exhaustion & Fatigue Scale. Comput Hum Behav Rep. 2021;4:100119. ^6^ Checklist for the manuscript is available on Figshare: “STROBE checklist Zoom fatigue” at https://doi.org/10.6084/m9.figshare.25487410. Data are available under the terms of the Creative Commons Attribution 4.0 International license (CC-BY 4.0).
