# Supplementary material for: Zoom fatigue related to online learning among medical students in Thailand:  Prevalence, predictors, and association with depression
Source: F1000Res. 2024 Aug 27;13:617. Originally published 2024 Jun 11. [Version 2] doi: 10.12688/f1000research.146084.2 (PMC11364962; doi:10.12688/f1000research.146084.2)
Supplement: Peer review history

# Reviewer report: Reviewer response for version 2

**DOI:** 10.5256/f1000research.170098.r323401

**Published:** 2024-10-03

**Authors:** Aidos Bolatov

**Affiliations:** 1 Astana Medical University, Astana, Kazakhstan

The paper titled "Zoom fatigue related to online learning among medical students in Thailand: Prevalence, predictors, and association with depression" investigates the prevalence of Zoom fatigue, its contributing factors, and its relationship with depression in a sample of Thai medical students. The authors conducted a cross-sectional survey using the Patient Health Questionnaire-9 and the Thai version of the Zoom Exhaustion & Fatigue Scale. The study identified that 9.6% of participants experienced high levels of Zoom fatigue, with a significant correlation between Zoom fatigue and depression.

 This paper addresses a timely and relevant topic, especially in the context of post-pandemic education. The study is well-designed and provides valuable insights into the mental health challenges faced by medical students during online learning. However, there are several areas where the paper could be strengthened:

 1. The methods section needs to be expanded. First, specify how all the variables presented in Table 1 were evaluated.

 2. Was it known which underlying disease the respondents had, were there any respondents with depression or other mental conditions among them?

 3. Please provide data on the internal consistency (cronbach's alpha) of the scales used in the study.

 4. For table 4, please provide complete statistical analysis data (chi-squared value).

 5. It would also be important to assess the correlation between levels of Zoom exhaustion (with all subscales) and depression.

 6. Regarding Table 5, it would be more correct to present all the variables (gender, both female and male, also for sleep, and so on), or specify in a more accessible way using "ref" or "vs". And also add 95%CI values for the coefficient.

 7. Another limitation of the study is the self-reporting nature of the questionnaire, which may introduce bias in the responses. Participants could have consciously or unconsciously misrepresented their answers, which can affect the accuracy and reliability of the data collected.

Is the work clearly and accurately presented and does it cite the current literature?

Yes

If applicable, is the statistical analysis and its interpretation appropriate?

Yes

Are all the source data underlying the results available to ensure full reproducibility?

Yes

Is the study design appropriate and is the work technically sound?

Yes

Are the conclusions drawn adequately supported by the results?

Yes

Are sufficient details of methods and analysis provided to allow replication by others?

Partly

Reviewer Expertise:

mental health, medical education, genetics, social psychology

I confirm that I have read this submission and believe that I have an appropriate level of expertise to confirm that it is of an acceptable scientific standard, however I have significant reservations, as outlined above.

# Reviewer report: Reviewer response for version 2

**DOI:** 10.5256/f1000research.170098.r317690

**Published:** 2024-08-30

**Authors:** Jonathan Salim

**Affiliations:** 1 Kalideres District General Hospital, West Jakarta, Jakarta, Indonesia

Thank you for the authors' response.

 Authors have satisfactorily answered the previous comments.

 However, it would be nice if the duplication issue although minor could be added to the limitation section.

 Please also elaborate on why the protocol ethic was approved in late January yet the data sampling begin in January. Does it begin before the approval is even approved?

 Thank you.

Is the work clearly and accurately presented and does it cite the current literature?

Yes

If applicable, is the statistical analysis and its interpretation appropriate?

Yes

Are all the source data underlying the results available to ensure full reproducibility?

Yes

Is the study design appropriate and is the work technically sound?

Yes

Are the conclusions drawn adequately supported by the results?

Yes

Are sufficient details of methods and analysis provided to allow replication by others?

Yes

Reviewer Expertise:

NA

I confirm that I have read this submission and believe that I have an appropriate level of expertise to confirm that it is of an acceptable scientific standard.

# Response

**Published:** 2024-09-13

**Authors:** Thammanard Charernboon

**Affiliations:** Department of Psychiatry, Thammasat University, Bangkok, Bangkok, Thailand

Thank you for your valuable feedback.

 1. Duplication Issue: We appreciate your suggestion and will add the duplication issue to the limitations section in the next version of the manuscript.

 2. Ethics Approval: Regarding the timing of data collection, we confirm that no data collection began before the ethics approval was granted. The approval was received on January 26, 2022, and we commenced survey distribution shortly thereafter, at the end of January 2022.

# Reviewer report: Reviewer response for version 1

**DOI:** 10.5256/f1000research.160123.r291515

**Published:** 2024-07-11

**Authors:** Jonathan Salim

**Affiliations:** 1 Kalideres District General Hospital, West Jakarta, Jakarta, Indonesia

Thank you for the opportunity to review this Zoom Fatigue article on the F1000 platform.

 The article corroborates that excessive online learning using Zoom or similar apps can exacerbate or induce student fatigue. It may lead to mental issues such as depression.

 Online learning and Zoom fatigue have become an essential topic to be discussed since the lockdown of the pandemic. However, some improvements are required to be addressed:

 1. The article needs more reasons why it should be done in the first place, or simply the research gap. Reproducing the ZEF questionnaire into many languages and producing a paper just because of differences in language would result in over a hundred papers on the same thing.

 2. Why the sample used in this study is medical students? Please address the sample selection and limited generalisability.

 3. Please elaborate on what you mean by "academic year 2565". Is the academic year in Thammasat different from the calendar year?

 4. Why are there no criteria for zoom usage in the participants' inclusion criteria? Adult students who just enrolled in the university and are proficient in Thai but do not do any Zoom online learning then can be included as study samples, which can lead to bias

 5. The study did account for missing data by making the questions required, yet did the authors filter for duplicates? since the survey was distributed through a lot of different medium. One student may fill in the survey from both the LINE group and Facebook group for example.

 6. It would be nice if the authors could provide how the ZEF questionnaire was translated into the Thai version. An invalid or unreliable translation method or procedure may generate invalid results.

 7. English language improvements are needed in some areas for an increase in readability and coherency.

 Thank you.

Is the work clearly and accurately presented and does it cite the current literature?

Yes

If applicable, is the statistical analysis and its interpretation appropriate?

Yes

Are all the source data underlying the results available to ensure full reproducibility?

Yes

Is the study design appropriate and is the work technically sound?

Yes

Are the conclusions drawn adequately supported by the results?

Yes

Are sufficient details of methods and analysis provided to allow replication by others?

Yes

Reviewer Expertise:

Medical Research and Application; Epidemiology; NTDs

I confirm that I have read this submission and believe that I have an appropriate level of expertise to confirm that it is of an acceptable scientific standard, however I have significant reservations, as outlined above.

# Response

**Published:** 2024-08-06

**Authors:** Thammanard Charernboon

**Affiliations:** Department of Psychiatry, Thammasat University, Bangkok, Bangkok, Thailand

**Reviewer 2**

 Thank you for your comments. We have responded to all of your concerns and hope our revisions address them comprehensively.

**Reviewer Comment:**

 Online learning and Zoom fatigue have become an essential topic to be discussed since the lockdown of the pandemic. However, some improvements are required to be addressed:

 1. The article needs more reasons why it should be done in the first place, or simply the research gap. Reproducing the ZEF questionnaire into many languages and producing a paper just because of differences in language would result in over a hundred papers on the same thing.

**Author Response:** Thank you for your comments. We have responded to all of your concerns and hope our revisions address them comprehensively.

 I have added to and rewritten our introduction as follows:

 “Due to its relatively recent emergence, Zoom fatigue has been the subject of limited research. Studies specifically focusing on medical students are even scarcer. Medical students differ significantly from other student populations, undergoing a six-year curriculum that includes clinical practice and often demanding on-call shifts. Understanding the risk factors for Zoom fatigue in this population could inform tailored prevention strategies and potentially mitigate negative impacts on academic performance and clinical decision-making.

 During the COVID-19 pandemic, all lectures at our university were delivered online, exposing the entire medical student body to this learning modality. To our knowledge, there are still only a few studies examining the prevalence of Zoom fatigue, its risk factors, and its potential association with depression, with no such study conducted among medical students in Thailand. To fill this knowledge gap, our study aims to assess the prevalence of Zoom fatigue in medical students during the COVID-19 pandemic, identify associated potential risk factors, and investigate its correlation with depression.”

**Reviewer Comment:**

 2. Why the sample used in this study is medical students? Please address the sample selection and limited generalisability.

**Author Response: **We focused on medical students because this program is demanding, spans six years, and includes clinical practice, which differentiates this sample from those in other faculties. We acknowledge this limitation in generalizability and have addressed it in the discussion as follows: “However, certain limitations must be acknowledged. Participants were recruited from a single site, which may limit the generalizability of the findings, and the results might not be applicable to other faculties.”

**Reviewer Comment:**

 3. Please elaborate on what you mean by "academic year 2565". Is the academic year in Thammasat different from the calendar year?

**Author Response: **We have removed this sentence to reduce confusion. We now describe the study period as follows: “A cross-sectional online survey was conducted among medical students at Thammasat University, Thailand, from January to July 2022.”

**Reviewer Comment:**

 4. Why are there no criteria for zoom usage in the participants' inclusion criteria? Adult students who just enrolled in the university and are proficient in Thai but do not do any Zoom online learning then can be included as study samples, which can lead to bias

**Author Response: **The study was conducted during the COVID-19 pandemic when the university mandated the use of videoconferencing for all lectures. Therefore, we believe that all participants were exposed to online learning. We have added this information to the Introduction section as follows: “During the COVID-19 pandemic, all lectures at our university were delivered online in compliance with government regulations, exposing the entire medical student body to this learning modality.”

**Reviewer Comment:**

 5. The study did account for missing data by making the questions required, yet did the authors filter for duplicates? since the survey was distributed through a lot of different medium. One student may fill in the survey from both the LINE group and Facebook group for example.

**Author Response:** We did not check for the duplication of participants. Due to the confidentiality of the survey, we did not record names, surnames, or other identifying information of the participants. However, since there is no incentive for the survey, we expect that duplication should be very low.

**Reviewer Comment:**

 6. It would be nice if the authors could provide how the ZEF questionnaire was translated into the Thai version. An invalid or unreliable translation method or procedure may generate invalid results.

**Author Response:** The Thai version of the ZEF was translated using the forward and backward translation method and was examined for psychometric properties, including factor validity, convergent validity, internal consistency, and test-retest reliability, in our previous publication. We have added this sentence to the manuscript: “The Thai version of the ZEF was developed using forward and backward translation. The details and other psychometric properties are presented in Charoenporn and Charernboon (2023).”

**Reviewer Comment:**

 7. English language improvements are needed in some areas for an increase in readability and coherency.

**Author Response:** We have rechecked and edited the manuscript with the help of our English speaker and ChatGPT-4.

 Thank you.

# Reviewer report: Reviewer response for version 1

**DOI:** 10.5256/f1000research.160123.r291510

**Published:** 2024-06-26

**Authors:** Pichai Ittasakul

**Affiliations:** 1 Department of Psychiatry, Faculty of Medicine, Ramathibodi Hospital,, Mahidol University, Bangkok, Thailand

I am writing to provide my opinion regarding the research paper I was invited to review for F1000Research. The paper explores the prevalence of Zoom fatigue related to online learning among medical students and identifies associated factors and correlations with depression in this population.

 I find the topic highly relevant and interesting, particularly in light of the increased use of Zoom for teaching during the COVID-19 pandemic in Thailand. This research contributes valuable insights into medical education and the mental health of medical students.

 Overall, the paper is well-written and presents its findings clearly. However, I recommend that the authors address the limitations regarding the generalizability of their findings, as the sample was collected from a single medical school in Thailand. Including this discussion would enhance the robustness of the study.

 Thank you for the opportunity to review this paper.

Is the work clearly and accurately presented and does it cite the current literature?

Yes

If applicable, is the statistical analysis and its interpretation appropriate?

Yes

Are all the source data underlying the results available to ensure full reproducibility?

Yes

Is the study design appropriate and is the work technically sound?

Yes

Are the conclusions drawn adequately supported by the results?

Yes

Are sufficient details of methods and analysis provided to allow replication by others?

Yes

Reviewer Expertise:

Mood disorders, Psychiatry

I confirm that I have read this submission and believe that I have an appropriate level of expertise to confirm that it is of an acceptable scientific standard.

# Response

**Published:** 2024-08-06

**Authors:** Thammanard Charernboon

**Affiliations:** Department of Psychiatry, Thammasat University, Bangkok, Bangkok, Thailand

**Author Response: **Thank you for the comment. We have added the limitation as follows:

 “This study represents the initial exploration of the adverse effects of online learning on medical students in Thailand, revealing a potential connection between Zoom fatigue and depression. However, certain limitations must be acknowledged. Participants were recruited from a single site, which may limit the generalizability of the findings, and the results might not be applicable to other faculties.”
